# Predictive Value of HAS-BLED Score Regarding Bleeding Events and Graft Survival following Renal Transplantation

**DOI:** 10.3390/jcm11144025

**Published:** 2022-07-12

**Authors:** Hans Michael Hau, Markus Eckert, Sven Laudi, Maria Theresa Völker, Sebastian Stehr, Sebastian Rademacher, Daniel Seehofer, Robert Sucher, Tobias Piegeler, Nora Jahn

**Affiliations:** 1 Department of Visceral, Transplantation, Vascular and Thoracic Surgery, University Hospital of Leipzig, 04103 Leipzig, Germany; hans-michael.hau@uniklinikum-dresden.de (H.M.H.); sebastian.rademacher@medizin.uni-leipzig.de (S.R.); daniel.seehofer@medizin.uni-leipzig.de (D.S.); robert.sucher@medizin.uni-leipzig.de (R.S.); 2 Department of Visceral, Thoracic and Vascular Surgery, University Hospital and Faculty of Medicine Carl Gustav Carus, Technische Universität Dresden, 01307 Dresden, Germany; 3 Department of Anesthesiology and Intensive Care Medicine, University Hospital of Leipzig, Liebigstrasse 20, 04103 Leipzig, Germany; sven.laudi@medizin.uni-leipzig.de (S.L.); mariatheresa.voelker@medizin.uni-leipzig.de (M.T.V.); sebastian.stehr@medizin.uni-leipzig.de (S.S.); tobias.piegeler@medizin.uni-leipzig.de (T.P.)

**Keywords:** kidney transplantation, anticoagulation, antiplatelet therapy, HAS-BLED score, cardiovascular disease

## Abstract

Objective: Due to the high prevalence and incidence of cardio- and cerebrovascular diseases among dialysis-dependent patients with end-stage renal disease (ERSD) scheduled for kidney transplantation (KT), the use of antiplatelet therapy (APT) and/or anticoagulant drugs in this patient population is common. However, these patients share a high risk of complications, either due to thromboembolic or bleeding events, which makes adequate peri- and post-transplant anticoagulation management challenging. Predictive clinical models, such as the HAS-BLED score developed for predicting major bleeding events in patients under anticoagulation therapy, could be helpful tools for the optimization of antithrombotic management and could reduce peri- and postoperative morbidity and mortality. Methods: Data from 204 patients undergoing kidney transplantation (KT) between 2011 and 2018 at the University Hospital Leipzig were retrospectively analyzed. Patients were stratified and categorized postoperatively into the prophylaxis group (group A)—patients without pretransplant anticoagulation/antiplatelet therapy and receiving postoperative heparin in prophylactic doses—and into the (sub)therapeutic group (group B)—patients with postoperative continued use of pretransplant antithrombotic medication used (sub)therapeutically. The primary outcome was the incidence of postoperative bleeding events, which was evaluated for a possible association with the use of antithrombotic therapy. Secondary analyses were conducted for the associations of other potential risk factors, specifically the HAS-BLED score, with allograft outcome. Univariate and multivariate logistic regression as well as a Cox proportional hazard model were used to identify risk factors for long-term allograft function, outcome and survival. The calibration and prognostic accuracy of the risk models were evaluated using the Hosmer–Lemshow test (HLT) and the area under the receiver operating characteristic curve (AUC) model. Results: In total, 94 of 204 (47%) patients received (sub)therapeutic antithrombotic therapy after transplantation and 108 (53%) patients received prophylactic antithrombotic therapy. A total of 61 (29%) patients showed signs of postoperative bleeding. The incidence (*p* < 0.01) and timepoint of bleeding (*p* < 0.01) varied significantly between the different antithrombotic treatment groups. After applying multivariate analyses, pre-existing cardiovascular disease (CVD) (OR 2.89 (95% CI: 1.02–8.21); *p* = 0.04), procedure-specific complications (blood loss (OR 1.03 (95% CI: 1.0–1.05); *p* = 0.014), Clavien–Dindo classification > grade II (OR 1.03 (95% CI: 1.0–1.05); *p* = 0.018)), HAS-BLED score (OR 1.49 (95% CI: 1.08–2.07); *p* = 0.018), vit K antagonists (VKA) (OR 5.89 (95% CI: 1.10–31.28); *p* = 0.037), the combination of APT and therapeutic heparin (OR 5.44 (95% CI: 1.33–22.31); *p* = 0.018) as well as postoperative therapeutic heparin (OR 3.37 (95% CI: 1.37–8.26); *p* < 0.01) were independently associated with an increased risk for bleeding. The intraoperative use of heparin, prior antiplatelet therapy and APT in combination with prophylactic heparin was not associated with increased bleeding risk. Higher recipient body mass index (BMI) (OR 0.32 per 10 kg/m^2^ increase in BMI (95% CI: 0.12–0.91); *p* = 0.023) as well as living donor KT (OR 0.43 (95% CI: 0.18–0.94); *p* = 0.036) were associated with a decreased risk for bleeding. Regarding bleeding events and graft failure, the HAS-BLED risk model demonstrated good calibration (bleeding and graft failure: HLT: chi-square: 4.572, *p* = 0.802, versus chi-square: 6.52, *p* = 0.18, respectively) and moderate predictive performance (bleeding AUC: 0.72 (0.63–0.79); graft failure: AUC: 0.7 (0.6–0.78)). Conclusions: In our current study, we could demonstrate the HAS-BLED risk score as a helpful tool with acceptable predictive accuracy regarding bleeding events and graft failure following KT. The intensified monitoring and precise stratification/assessment of bleeding risk factors may be helpful in identifying patients at higher risks of bleeding, improved individualized anticoagulation decisions and choices of antithrombotic therapy in order to optimize outcome after kidney transplantation.

## 1. Introduction

Patients with chronic kidney disease (CKD) listed for kidney transplantation (KT) present with a high prevalence and incidence of cardio- and cerebrovascular diseases, requiring antiplatelet and/or anticoagulant treatment [1]. The management of peri- and postoperative anticoagulation as well as antiplatelet regime in this high-risk patient population becomes increasingly challenging due to an increased risk of both thrombotic and hemorrhagic postoperative complications [2,3]. In this context, the risk of serious perioperative thromboembolic complications must be weighed against the risk of bleeding events after KT.

For patients with end-stage renal disease (ESRD) scheduled for KT, the half-life of anticoagulant/antiplatelet drugs may become a perioperative challenge [1]. The tightly restricted timeframe of (deceased donor) transplantation prevents the discontinuation of medication within a reasonable period before transplantation. Surgery then often must proceed despite recent antiplatelet therapy (APT) and/or anticoagulant medication intake, increasing the risk of perioperative bleeding as well as the risk for impaired early and long-term graft function and patient outcome. To date, no consensus or evidence-based protocols for antithrombotic therapy and its management following KT are available. Most previous studies focus on the investigation of the effects and benefits of antithrombotic prophylaxis and therapy for the prevention of postoperative thromboembolic complications after KT [4,5,6,7,8]. However, the risks of bleeding and particularly of modifiable risk factors in this vulnerable patient collective are less clearly described [4,5]. To facilitate the prediction of patients’ individual bleeding hazards on anticoagulants, various risk stratification and assessment tools have been developed in the past, including the most prominent ones such as the (modified) Outpatient Bleeding Risk Index (m-OBRI), HAS-BLED score, ATRIA, HEMORR2HAGES, ABC bleeding risk score, RIETE risk scheme, ORBIT and QBLEED score ([9,10,11,12,13,14,15,16]). In summary, these tools are trying to assess—and if possible, reduce—the modifiable risk factors for bleeding events (e.g., blood pressure range, specific coagulation measurement and discontinuation/resumption of anticoagulant drugs) as well as to assist clinicians and patients in making informed and individualized anticoagulation decisions and choices of antithrombotic therapy. However, the different developed assessment tools vary significantly in the predictive accuracy and choice of individual risk components, the definition and emphasis of clinical factors as well as their integration in clinical practice [17,18,19]. To date, the most accurate, well-evaluated and confirmed assessment tool is the HAS-BLED score, implementing arterial hypertension, abnormal renal and liver function, stroke, bleeding history or predisposition, labile international normalized ratio, elderly (>65 years) and drugs/alcohol concomitantly to predict the risk of major bleeding during anticoagulant (the use of vitamin K antagonists) or antiplatelet therapy [10,20]. However, detailed insight in risk–benefit analysis and the influence of applying the HAS-BLED score in patients after KT on graft and patient outcome and function have not yet been described. 

Therefore, the purpose of our current study was to assess the effect of different antithrombotic management strategies on the incidence of bleeding and successive patient and graft function and outcome following KT. Additionally, a special focus was placed on clinical risk stratification as well as the usefulness and predictive value of the HAS-BLED score on the prediction of postoperative bleeding and early and long-term outcome of KT recipients.

## 2. Material and Methods

### 2.1. Study Design and Study Population

The study protocol was approved by the local ethics committee of the University of Leipzig (protocol number: 111-16-14032016). From a prospectively collected electronic database, we retrospectively analyzed medical data of all patients undergoing kidney transplantation (KT) at the University Hospital of Leipzig, Germany, between 2011 and 2018. A special focus was set on peri- and postoperative antithrombotic therapy with incidence of bleeding episodes as well as validation of clinical bleeding scores (specifically the HAS-BLED score) with regard to postoperative early and long-term graft and patient function and outcome. Patients younger than 18 years, with combined transplantations and/or dual KT or with a lack of data on antithrombotic therapy as well as patients with insufficient hospital records were excluded from the analysis. 

### 2.2. Study Groups/Antithrombotic Strategy

According to their pretransplant anticoagulation therapy, patients were stratified and categorized into the prophylaxis group (group A)—patients without pretransplant anticoagulation/antiplatelet therapy and receiving postoperative heparin in a prophylactic dose (n = 108 patients)—and into the (sub)therapeutic group (group B)—patients with postoperative continued use of (sub)therapeutically antithrombotic medication (n = 96 patients). This antithrombotic therapy consisted of heparin in therapeutical dosage (n = 22), the continued use of vitamin K antagonists (VKA) or direct oral anticoagulants (DOAC) (n = 8) at the day of transplantation, antiplatelet therapy (APT) and heparin in prophylactic doses (n = 53) and APT and heparin in therapeutical doses (n = 13) (Table S1). Continued use of VKA was defined as preoperative INR > 1.5, which was not altered preoperatively and with the last administration of VKA at the day of transplantation. Continued used of DOAC was defined with the last administration of DOAC at day of transplantation. All these patients received correction for international normalized ratio (INR) by vitamin K or prothrombin complex concentrate after admission to the hospital and then were bridged with unfractionated heparin. Patients whose VKA therapy was bridged with heparin before transplantation (living kidney donation, medical problems, etc.) were included in the VKA group. These patients were treated after KT with heparin in a therapeutical dose. Therapeutical heparin use was monitored by partial thromboplastin time (PTT) measurement within a range of 40–60 s in our center. 

### 2.3. Definition of Bleeding Episodes 

Postoperative bleeding after KT was defined as a decrease in hemoglobin levels of more than 2 g/dL over 24 h with the need for blood transfusion and/or intervention (surgery or drainage) and/or a subsequent ultrasonography (specifically 1 day prior to 2 days following the significant hemoglobin drop) identifying a hematoma or large fluid collection, comparably to methods described previously [7]. Hematoma detected by ultrasound was only determined to be relevant if it required transfusion or other interventions.

### 2.4. Outcome Measures and Analysis

Standard demographic and clinical characteristics were collected and analyzed before, at the time of and after transplantation in the follow-up period for each patient: the pretransplantation data included recipient and donor characteristics such as age, sex, body mass index (BMI), donor’s causes of death, and donor’s comorbidities/clinical course. Furthermore, recipient data included the presence of diabetes mellitus, time on the waiting list, the type and duration of pretransplantation dialysis, metabolic endocrine and lipid metabolism, preoperative platelet count and INR, American Society of Anesthesiologists (ASA) physical status score, information on the cause of ESRD and recipient’s comorbidities (presence of coronary heart disease, peripheral arterial disease (PAD) and arterial hypertension as well as the number of antihypertensive agents) as well as current and prior anticoagulation medication and history. As previously described, the HAS-BLED score was used to predict bleeding in patients under vitamin K antagonists and antiplatelet therapy [10]. 

Assessed peri- and postoperative data included information on peri- and postoperative clinical course (operation time, blood loss and cold and warm ischemia time), intraoperatively administered amounts of fluids and blood products (fresh frozen plasma (FFP), erythrocyte concentrates (EC)), incidence of bleeding and thromboembolic complications (TEC), acute rejection episodes (ARE), delayed graft function (DGF) as well as surgical and infectious complications. Rejection episodes were, whenever clinically feasible, histologically confirmed. Treatment of acute cellular rejection consisted of pulsed steroids or administration of antithymocyte globulin (ATG, 8 mg/kg bodyweight) along with increased baseline immunosuppression. DGF was defined as the requirement of dialysis in the first week following transplantation [21]. Postoperative complications occurring during the first three months after transplantation were noted. Graft failure was defined as patient death, return to dialysis or retransplantation (patients who died before graft failure were censored).

Further data included immunological and immunosuppressive characteristics (human leukocyte antigen (HLA) mismatches, cytomegalovirus (CMV) state and induction therapy) as well as patient and graft function and outcome. Complications were rated according to the Clavien–Dindo classification (CDC) and dichotomized into minor (CDC 0–2) and severe (CDC 3–5) complications [22]. Renal function and outcome (GFR, creatinine (mmol/L) and urea (mmol/L)) were analyzed in the follow-up period after transplantation.

### 2.5. Surgical Techniques/Intraoperative Antithrombotic Protocol

As described previously, kidney grafts were procured and transplanted following international standards and guidelines [23,24,25,26,27]. In short, kidney grafts were transplanted retroperitoneally into the iliac fossa. Vascular anastomoses were performed on the common or external iliac artery and vein. In cases of multiple arterial reconstructions, an end-to-end or end-to-side anastomosis was performed. In some deceased donor organs, attachment to the aortic patch was existing and thus extensive arterial reconstruction was not needed. In some cases, a second additional renal artery was too small, so it was sacrificed. The ureter was implanted into the bladder according to the Lich–Gregoir technique using a double J catheter as an intraurethral splint [28]. Splint removal was performed 3–4 weeks after successful transplantation. 

According to our center protocol, patients received 5000 IU unfractionated heparin (UFH) intravenously before crossclamping of the iliac vessels. In recipients receiving kidney transplants where reconstruction of multiple arteries was necessary, continuous UFH with a starting dose of 15 IU/kg × body weight was applied and adapted to PTT values of 40–60 s with PTT measurements every 4–6 h, starting 6 h postoperatively. All patients of group A (prophylaxis group) received UFH in prophylactic doses of 200–400 IE/h as part of hospital protocol for immobilized patients, starting 6 h postoperatively. If post-transplantation GFR permanently increased above 30 mL/min, continuous UFH was replaced by low-molecular-weight heparin (tinzaparin (Innohep^®^): 0.35 mL, 3.500 IE ant-XA or nadroparin (Fraxiparine^®^): 0.3 mL, 2.850 I.E. anti-Xa) subcutaneously once daily.

### 2.6. Immunosuppression

Immunosuppressive therapy comprised an induction therapy with the interleukin-2 receptor antagonist basiliximab or antithymocyte globulin (ATG), followed by a triple maintenance immunosuppression consisting of calcineurin inhibitors (tacrolimus or cyclosporine) and/or antimetabolites (mycophenolate mofetil or sirolimus) and tapered steroids (prednisolone) [29]. 

### 2.7. Statistical Analysis

With regard to baseline data, descriptive statistics are presented as mean values with standard deviation for continuous variables and were analyzed by the Student’s *t*-test, analysis of variance (ANOVA) and the Mann–Whitney U or Kruskal–Wallis tests, depending on distribution, which in turn was evaluated by a Shapiro–Wilk test. Categorical variables are presented as totals with percentages (n (%)). Differences between patients with or without complications after transplantation were evaluated by chi-square and/or Fisher’s exact tests.

For the determination of a possible relationship between antithrombotic therapy and the incidence of bleeding, univariate and multivariate logistic regression models were used. Under the multivariate model, potential risk factors with significant *p*-values in the univariate analysis (*p* < 0.05) and/or known risk factors from the literature were selected using the backward stepwise selection procedure with adjustment for potential confounders. Results of the regression analyses are presented as odds ratio (OR) with 95% confidence interval (CI) and its corresponding *p*-value. Hoshmer–Lemshow test (HLT) was used to test fitness and calibration of the models. The model was considered fit when *p* > 0.05. Harrell’s C-index and the area under the curve (AUC) of the receiver operating characteristic curve (ROC) were used to calculate the discriminative power and prognostic accuracy of the model. 

For the survival outcome analyses, the Kaplan–Meier method was used and a log-rank test was applied to test statistical significance between the different analyzed groups. The time origin for the bleeding analysis was date of bleeding and all-cause graft loss (defined as return to dialysis, retransplantation or death). 

Uni- and multivariate Cox proportional hazard regression models were used to calculate hazard ratios (HR) with 95% confidence intervals (CI) for all-cause graft loss. For variables to be entered into the multiple logistic regression analysis, we used a backward stepwise regression model including clinically and paraclinically relevant variables which were chosen on the basis of the results of univariate analysis (*p* < 0.05). Sensitivity analyses as well as C-statistics/AUC models were performed to test the robustness of the main results. All statistical analyses were performed using SPSS software (SPSS Inc., Chicago, IL, USA, version 21.0). *p*-values less than 0.05 were considered to be statistically significant. 

## 3. Results

### 3.1. Baseline Characteristics

A total of 204 consecutive patients undergoing KT at our institute between 2011 and 2018 were included in our study with a mean follow-up period of 7.1 ± 2.20 years. In total, 53% of our patients received postoperative anticoagulation with heparin in a prophylactic dose (group A), whereas 47% received a postoperative antithrombotic regime in a (sub)therapeutical dose (group B).

Baseline and clinical characteristics and pre- and perioperative parameters stratified for the different antithrombotic medication schemes are illustrated in Table 1. An elevated rate of performed pre-emptive transplantations was observed in the prophylaxis group (group A) (*p* = 0.045), whereas increased age (*p* = 0.015) and rates of cardiovascular comorbidities were more evident in the (sub)therapeutic group (group B) (*p* <0.01). The HAS-BLED score was 2.17 +/− 0.69 in the prophylaxis group (group A) compared to 3.22 +/− 1.08 (*p* < 0.01) in the (sub)therapeutic group (group B). All other baseline characteristics were comparable between both groups without significant differences.

### 3.2. Incidence of Bleeding 

**Figure 1:**
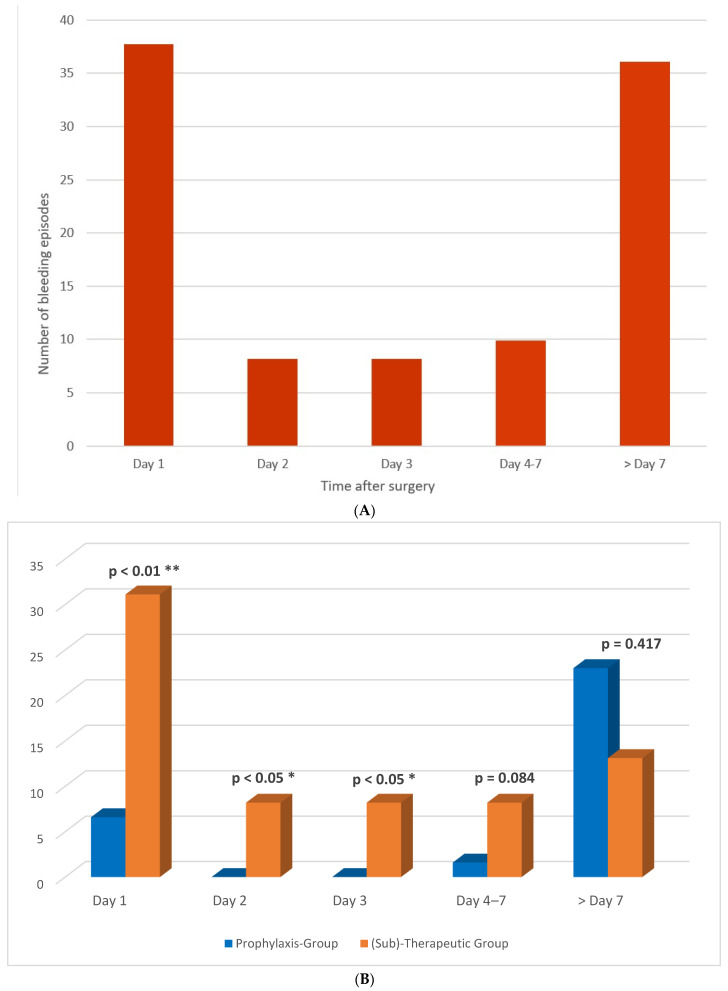
(**A**)**.** Total number of bleeding episodes (in percent) per day of all 204 patients following kidney transplantation. (**B**). Number of bleeding episodes (in percent) per day stratified by antithrombotic therapy regime after kidney transplantation. *p* < 0.05 *; *p* < 0.01 **.

The baseline and clinical characteristics stratified for the bleeding status are illustrated in Table 2. Postoperative bleeding occurred in 29.9% of all patients, mean time to bleeding was 7.1 ± 8.2 days (n = 22; 36.1%) with a peak on day 1 (n = 23; 37.7%), and one-third of the bleeding events occurring in the late phase after 7 days (n = 22 patients; 36%) (Figure 1A). In total, 64% of the bleeding episodes occurred in the first 7 days. An amount of 21 (34%) bleeding incidents occurred in the prophylaxis group and 40 (66%) in the (sub)therapeutic group (Figure 1B). From the latter mentioned (sub)therapeutic group, 5 (13%) cases of bleeding occurred in the VKA group, 9 (15%) in the therapeutically heparin group, 17 (28%) in the antiplatelet and prophylactic heparin group and 9 (15%) in the antiplatelet and therapeutically heparin group. The incidence (*p* < 0.01) and timepoint of bleeding over the postoperative days (*p* < 0.01) varied significantly between the different treatment groups (Figure 1B). The presence of cardiovascular disease (n= 34; 55.7%; *p* < 0.01) and the prior use of anticoagulation (n = 36; 59%; *p* < 0.01) were significantly increased in the bleeding group, baseline platelets (*p* = 0.01) were significantly lower in the bleeding group compared to that of the nonbleeding. The HAS-BLED score was 3.25 ± 1.2 in the bleeding group compared to 2.5 ± 0.9 in the nonbleeding group (*p* < 0.01). Of the 61 patients with postoperative bleeding, 43 required surgical and/or interventional procedures with or without transfusion, 9 required only transfusion and in 9 expectant management in the sense of “watch and wait strategy” was performed.

### 3.3. Intra- and Postoperative Outcome

Intra- and postoperative graft function and outcome variables stratified according to the antithrombotic medications are shown in Table 3. The amount of total transfusion volume (*p* = 0.024) as well as the transfusion of erythrocyte concentrates (*p* = 0.044) were increased in the (sub)therapeutic group. On the other hand, patients in the prophylaxis group showed an increased diuresis volume at 1 h postreperfusion (162 ± 92 versus 68 ± 101, *p* = 0.042) and at day 1 (1961 ± 1290 versus 1240 ± 1100; *p* < 0.01) compared to patients in the (sub)therapeutic medication group. The mean time until the initiation of heparin after transplantation was 6.7 ± 3.1 h in the prophylaxis group compared to 5.5 h ± 3.8 in the (sub)therapeutic group (*p* = 0.017). Significant differences in coagulation parameters with regard to thromboplastin time and PTT were observed at days 1, 3 and 5 following KT between both groups, whereas no significant differences were observed for platelets (Table 3). Regarding the postoperative outcome, the incidence of bleeding episodes (*p* < 0.01), the rate of delayed graft function (*p* < 0.01) as well as major (>grade II) complications according to CDC (*p* < 0.01) were significantly increased in the (sub)therapeutic group, whereas the incidence of thromboembolic events (*p* = 0.08) and the rate of rejection episodes (*p* = 0.098) showed no significant differences between the groups. 

The differences between the bleeding and nonbleeding groups concerning intra- and postoperative graft function and outcome variables are illustrated in Table 4. Herein, the duration of surgery (210 ± 72 min, *p* = 0.02), blood loss (274 ± 488 mL, *p* = 0.015) as well as the intraoperative demand of red blood cells (319.67 ± 672 mL *p* < 0.01) and platelets (19.67 ± 74.88 mL, *p* = 0.045) were increased in the bleeding group. Further, the rates of DGF (37.7%, *p* = 0.018), TECs (11%, *p* = 0.02) and major complications (>grade II) according to Clavien–Dindo (48%, *p* < 0.01) were significantly increased in the bleeding group. The 1 h postreperfusion diuresis was higher in the nonbleeding group (*p* = 0.048). Significant differences concerning coagulation variables PTT and platelet count could be observed at day 1 and 3 following KT. Notably, the mean time to the initiation of heparin administration after transplantation was significantly shorter in the bleeding group (5.3 ± 3.4 h after KT) compared to that in the nonbleeding group (6.5 ± 5.3 h after KT) (*p* = 0.028). In the bleeding group, in 17 patients (28%) antiplatelet therapy was started within 24 h after KT compared to 22 patients (15%) in the nonbleeding group. The start of antiplatelet therapy within 24 h after KT significantly correlated with the incidence of bleeding (r = 0.145; *p* = 0.038). However, the start of antiplatelet therapy 2 (r = 0.10; *p* = 0.148) or 3 days (r = 0.12; *p* = 0.09) after KT did not show any significant correlations with postoperative bleeding.

Concerning renal function and graft and patient outcome at one, three, six and twelve months following KT, no significant differences between the two antithrombotic regimes (prophylaxis group vs. (sub)therapeutic group) and between the bleeding and nonbleeding groups could be observed (Table S2). 

### 3.4. Risk Factors for Bleeding

In univariable analysis for potential risk factors for bleeding following KT (Table S3), the postoperative (sub)therapeutically antithrombotic medication regime (OR 2.95 (95% CI: 1.58–5.53); *p* < 0.01), postoperative therapeutical continuous heparin infusion (OR 3.95 (95% CI: 1.95–8.02); *p* < 0.01) and continued use of VKA (OR 6.25 (95% CI: 1.18–33.39); *p* = 0.018) increased the risk of postoperative bleeding. Intraoperative heparin use (OR 2.33 (95% CI: 0.89–6.07); *p* = 0.082) and preoperative antiplatelet therapy (OR 1.57 (95% CI: 0.82–2.81); *p* = 0.186) did not show any significant increases in bleeding risk. Donor age > 55 years was associated with a nearly two-fold increase in the risk of postoperative bleeding (OR 1.97 (95% CI: 1.07–3.62); *p* = 0.028) and every point increase in the ASA scale increased the risk of bleeding by 2.21 times (OR 2.21 (95% CI: 1.04–4.89); *p* = 0.028). Patients suffering from CVD had a 2.6-fold risk increase (OR 2.57 (95% CI: 1.39–4.75); *p* < 0.01) and every year on dialysis increased the risk by 1.13 times (OR 1.13 (95% CI: 1.06–1.22); *p* = 0.01). Every point increase in the HAS-BLED score increased the risk of bleeding by 1.67 times (OR 1.67 (95% CI: 1.23–2.23); *p* < 0.01). Intra- and postoperative variables and outcome factors such as blood loss (OR 1.01 (95% CI: 1.01–1.03); *p* = 0.04), the duration of surgery (OR 1.09 (95% CI:1.00–1.02); *p* < 0.01), catecholamine use (OR 8.12 (95% CI: 2.9–21.9); *p* < 0.01), infectious complications (OR 6.9 (95% CI: 2.67–17.82); *p* <0.01), rejection episodes (OR 6.81 (95% CI: 2.67–17.82); *p* < 0.01), procedure-specific complications based on CDC (OR 8.62, 95% CI: 4.05–18.4; *p* < 0.01) as well as the occurrence of delayed graft function (DGF) (OR 2.74 (95% CI: 1.45–5.08); *p* < 0.01) significantly increased the risk of postoperative bleeding. Interestingly, patients who received dialysis at POD 0 to 2 due to DGF had a significantly increased risk of bleeding compared to patients between POD 3 to 7 (OR 2.92 (95% CI: 1.52–5.59); *p* < 0.01). A second warm ischemia time (anastomosis time) >45 min (OR 0.38 (95% CI: 0.0.18–0.63); *p* < 0.01) and living kidney transplantation (OR 0.41 (95% CI: 0.18–0.92); *p* = 0.032) significantly decreased the risk of postoperative bleeding. Every increase of 5 kg/m^2^ in recipient BMI (OR 0.49 (95% CI: 0.21–0.99); *p* = 0.047) decreased the risk by more than a half and the start of anticoagulation therapy after 6 h decreased the risk by two-fifths (OR 0.51 (95% CI: 0.29–0.99); *p* = 0.045).

In a second analysis, calibration and prognostic accuracy as well as the predictive performance of the HAS-BLED score for predicting postoperative bleeding events were evaluated. The calibration of the model was good, demonstrated by the nonsignificant results of the HLT (*p*-value: 0.202, chi-square: 4.62). The predictive performance of the model for bleeding episodes was moderate, with an AUC of 0.72 (0.63–0.79; *p* < 0.01). The cut-off value was set at 3.5 (2.5) according to the ROC curve in our KT patient cohort, with a sensitivity and specificity of 49% (69%) and 83% (62%), respectively (Figure S1).

For multivariable-adjusted analyses according to the backward stepwise selection procedure and well-known risk factors from the literature, 22 key risk factors from the total pool of factors were analyzed in the multivariable model (Table 5). The Hosmer–Lemeshow test showed the fitness of the model (chi-square: 4.572; *p* = 0.802). The C-statistic of this model was 0.73 (0.69–0.76), indicating a good degree of discrimination and predictive performance. 

Pre-existing cardiovascular disease (CVD) (OR 2.89 (95% CI: 1.02–8.21); *p* = 0.04), blood loss (OR 1.03 (95% CI: 1.0–1.05); *p* = 0.014), HAS-BLED score (OR 1.49 (95% CI: 1.08–2.07); *p* = 0.018), Clavien–Dindo classification > grade II (OR 1.03 (95% CI: 1.0–1.05); *p* = 0.018), the continued use of VKA (OR 5.89 (95% CI: 1.10–31.28); *p* = 0.037), (sub)therapeutically antithrombotic medication therapy (OR 2.14 (95% CI: 1.08–4.24); *p* = 0.015) as well as postoperative heparin in therapeutical doses (OR 3.37 (95% CI: 1.37–8.26); *p* < 0.01) remained independently associated with risk for postoperative bleeding after KT. 

Preoperative antiplatelet therapy (OR 1.63 (95% CI: 0.85–3.15); *p* = 0.144), intraoperative heparin use (OR 1.35 (95% CI: 0.44–4.11); *p* = 0.597) and the start of antithrombotic medication therapy < 6 h (OR 1.58 (95% CI: 0.85–2.98); *p* = 0.151) did not show significant results. Higher recipient BMI (OR 0.32 per 5 kg/m^2^ increase in BMI (95% CI: 0.12–0.91); *p* = 0.023) as well as living donor KT (OR 0.43 (95% CI: 0.18–0.94); *p* = 0.036) were also further associated with decreased risk for bleeding.

After adjustment for antithrombotic therapy and subgroup analysis, the combination of APT and therapeutic heparin (OR 5.44 (95% CI: 1.33–22.31); *p* = 0.018) as well as postoperative therapeutic heparin (OR 3.37 (95% CI: 1.37–8.26); *p* < 0.01) were also further associated with an increased risk of bleeding.

#### Graft Outcome and Survival

**Figure 2:**
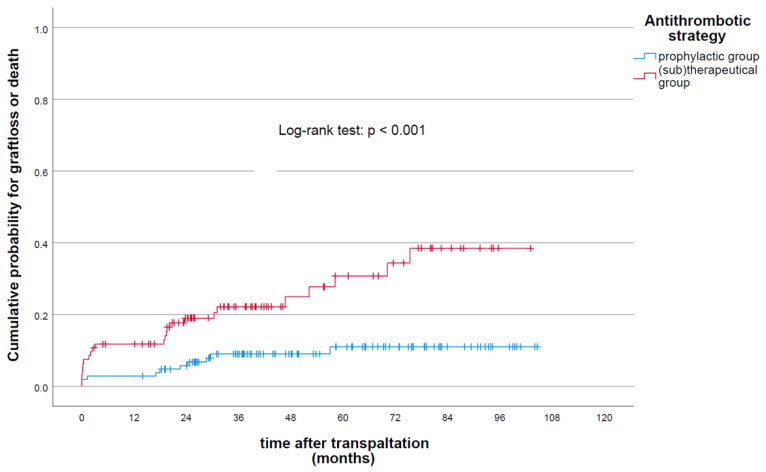
Cumulative probability of graft loss and/or death following renal transplantation stratified by antithrombotic regime.

Kaplan–Meier curves for graft loss or death stratified by antithrombotic treatment are shown in Figure 2. The cumulative probability of graft loss or death was significantly higher among patients receiving (sub)therapeutic antithrombotic therapy compared to the prophylactic group (*p* < 0.01). Specifically, one-, three-, five- and ten-year graft survival was 97.2%, 91%, 89.1% and 89.1% in the prophylactic group compared to 88.3%, 77.9%, 69.3% and 61.6% in the (sub)therapeutic group, respectively. Table 6 shows the Cox regression analysis of clinical and paraclinical predictors for graft loss following KT. In this context, patients receiving (sub)therapeutic antithrombotic therapy had a quantitively higher risk of graft loss or death in the multivariate final model, showing a HR of 2.9 ((95% CI: 1.41–6.23); *p* < 0.01). 

**Figure 3:**
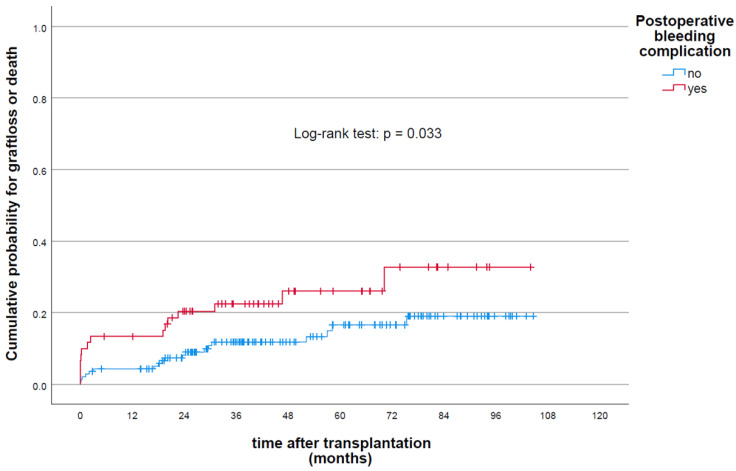
Cumulative probability of graft loss and/or death following renal transplantation stratified by bleeding status of the recipients.

The cumulative risk of graft loss and/or death was significantly higher among patients with postoperative bleeding compared to those of nonbleeding cases (log-rank test, *p* = 0.033) (Figure 3). Multivariate COX regression analysis in patients with bleeding versus nonbleeding showed a higher risk of graft loss and/or death (HR, 2.52, 1.1–5.1, *p* = 0.029). 

**Figure 4:**
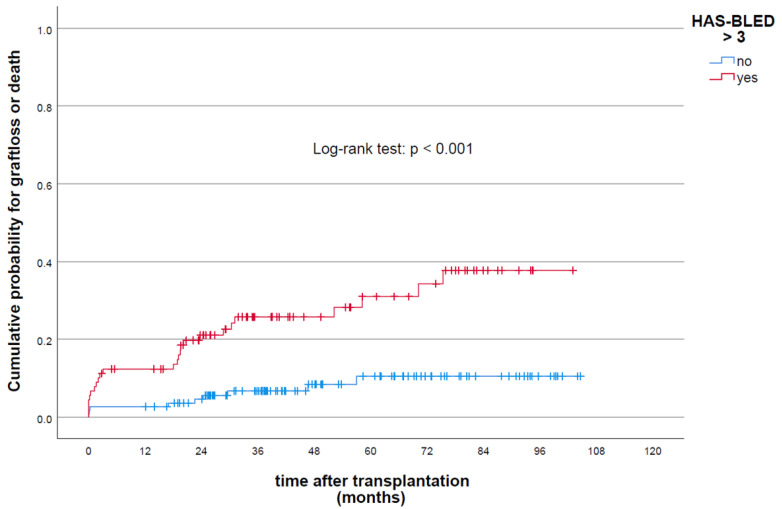
Cumulative probability of graft loss and/or death following renal transplantation stratified by HAS-BLED score.

According to HAS-BLED score stratification, patients with scores >3 showed a significantly increased risk of graft loss (HR 3.2 (95% CI: 1.46–7.1); *p* < 0.01) (Figure 4). With regard to the prediction of renal allograft failure, the HAS-BLED risk model, specifically with a cut-off value > 3, showed moderate prognostic accuracy (AUC 0.7 (95% CI: 0.61–0.78)) and calibration (HLT: chi-square: 6.52; *p* = 0.18). 

As further independent prognostic clinical and paraclinical (e.g., used pharmacological substances) predictors for graft loss, a donor age >55 years, a recipient history of diabetes, the duration of surgery, acute rejection episodes, a Clavien–Dindo score > 2, preoperatively continued anticoagulation therapy, postoperative catecholamine use as well as the combination of antiplatelet medication and therapeutical heparin use in the postoperative antithrombotic medication regime could be identified (Table 6).

When patients were stratified by the severity of bleeding, the cumulative probability of long-term graft loss/death was lowest in patients with no bleeding events as well as in patients who received adequate reoperations/interventions after incidents of bleeding compared to postoperative watchful waiting management with transfusion alone (*p* = 0.102) (Figure S2). The univariable hazard ratio for transfusion or reoperation/interventions (versus no bleeding) was significantly elevated (HR 2.03 (95% CI: 1.05–4.13); *p* = 0.048), compared to expectant management, which showed no significant values (HR 1.31 (95% CI: 0.75, 2.27); *p* = 0.291). 

When investigating kidney transplant outcomes among patients with versus without bleeding according to donor type (living versus deceased organ donation; Figure S3), the univariable hazard ratio associated with deceased donor kidney transplants was markedly elevated as compared to that associated with living donor kidney transplants (HR 2.5 (95% CI: 1.226–5.038) versus HR 0.96 (95% CI: 0.226–4.163); *p* = 0.035). 

## 4. Discussion

Our study shows that different antithrombotic treatment regimes including the use of VKA, postoperative therapeutic heparin and APT plus additional heparin in therapeutic dosages were strong independent predictors of increased postoperative bleeding and associated with a deterioration in graft and patient outcome. Regarding the evaluation of the predictive value of the HAS-BLED score, we were able to demonstrate an overall moderate predictive accuracy for the detection of postoperative bleeding episodes (AUC 0.72) and allograft failure (AUC 0.70) in our KT population. On the other hand, high BMI and living kidney donation showed a decreased risk of postoperative bleeding episodes. Interestingly, the use of intraoperative heparin, prophylactic anticoagulation as well as APT or a combination of both was not associated with an increased bleeding risk. Thus, our study indicates that the timing as well as type of anticoagulation and antiplatelet therapy seem critical for incidents of perioperative bleeding and subsequent graft and patient outcome after kidney transplantation.

In the clinical context of ESRD and consecutively of the renal transplant setting, thrombosis and bleeding risks are simultaneously increased and may have devasting consequences for perioperative graft and patient outcome [30,31]. While anticoagulation and antiplatelet drugs are often indispensable for the prevention of TECs in this high-risk patient population, the significantly increased risk of bleeding complicates the perioperative handling of these medications. Within the last years, independent clinical bleeding risk models—mainly based on historical bleeding variables in the literature—have been developed to predict the risk of major bleeding during the use of vitamin K antagonists, DOACs or antiplatelet therapy in a wide range of patients with a moderate predictive ability [17,18,19,32]. One of the best validated scores is the HAS-BLED score. However, data on validation and predicting bleeding risk with the HAS-BLED score in ERDS and KT recipients are lacking [10,20]. In our current study, we show for the first time that the HAS-BLED score, specifically a cut-off value > 3, was an independent predictor of postoperative bleeding and allograft failure in multivariable analysis in our collective of ESRD patients undergoing KT. Interestingly, this risk model demonstrates a good discriminative and predictive accuracy as well as good calibration for the prediction of bleeding episodes and renal allograft failure. Hence, the perioperative evaluation of the HAS-BLED score might be an interesting tool for risk assessment in patients receiving anticoagulation and antiplatelet drugs after KT. 

Although the time period, during which severe bleeding events were observed postoperatively, ranged from day 1 to day 21, most bleeding incidents (approximately 65%) were observed in the early postoperative period within the first 7 days after the initial operation, which is in accordance with the available literature [4,5,33,34,35]. However, approximately one-third of the bleeding episodes were observed in the late phase, here depicted cumulatively at and after day 7 after the transplantation (see Figure 1A). Possible reasons for the observed late bleeding events in our patient population are in accordance with previous observations, for instance, secondary systemic infections with deranged systemic coagulation, thromboembolic complications with the need of a new initiation of therapeutic heparin, an accumulation of molecularly low-weight heparin due to still-reduced kidney function as well as rejection episodes with a need of extracorporeal therapy with plasmapheresis and a concomitant increase in therapeutical systemic anticoagulation. Moreover, 7 days postoperatively, most patients on prior anticoagulant/antiplatelet medication were restarted on the original medication, which in some cases may also have added to the increased risk of bleeding. 

Nevertheless, in light of constant improvement in the postoperative management of kidney recipients, and especially for the early detection and considerate management of bleeding incidents, critical timeframes underlined by our observations may help to increase vigilance and improve outcomes in the postoperative recovery period. Moreover, our results may also indicate an interesting insight in the optimal timing of postoperative anticoagulation or/and antiplatelet readministration after kidney transplantation. The majority of patients with ESRD and hence recipients of kidney grafts have prescriptions of anticoagulative therapy, e.g., due to a history of thrombosis or stroke [36,37]. Prior to elective surgical procedures, the anticoagulative drugs are usually discontinued within an adequate time lag before surgery. However, due to the nature of the conditional limited time frame of deceased organ donation, the timely discontinuation of anticoagulative drugs is often not feasible [38]. In this context, the optimal timepoint for the postoperative resumption of anticoagulative medication following kidney transplantation is not clear. Our current data hint that an initiation of anticoagulation <6 h and an intake of antiplatelet therapy within 24 h after KT are significant predictors of the development of postoperative bleeding in univariable analysis and should possibly be avoided, whenever possible. In accordance with our results, other studies reported an increased risk for bleeding after a continuation of VKA in the perioperative period with urgent indications for continued VKA therapy in certain patients and risk constellations [5,39]. Likewise, in our current study, all patients on continued anticoagulative or antiplatelet therapy had vital indications for anticoagulation therapy, e.g., due to atrial fibrillation with high CHA2DS2-VASc scores, a history of deep venous thrombosis and/or pulmonary embolisms, a history of acute myocardial infarction with stenting of the coronary arteries or a history of stroke. In all these cases, the treating physicians were aware of the possible complications and deemed discontinuing anticoagulant medication perioperatively as more threatening than the risk of a postoperative bleeding. However, every patient receiving anticoagulative and/or antiplatelet medication in the perioperative setting, especially after organ transplantation, must be individually and critically evaluated on the risks and benefits of early reintroduction to anticoagulants. Our results emphasize that the untimely and inconsiderate initiation of anticoagulative drugs during this critical timeframe in the early postoperative period after kidney transplantation must be avoided.

Currently, there are, however, no specific guidelines in the postoperative antithrombotic management of renal transplant recipients, and a careful balance between the prevention of thromboembolic complications (TEC) and bleeding incidents should be the first important clinical goal. While the existing data for considering antithrombotic prophylaxis in KT are limited by their sample size, a clear answer to whether antithrombotic/antiplatelet therapy increases bleeding risk, as well as the optimal antithrombotic postoperative management with regard to graft outcome, is less clear. A previous study by Pawlicki et al. reported an association between an increased risk for bleeding and antithrombotic prophylaxis [7]. However, these results should be interpreted with caution due to the small sample size and suboptimal methodology in bleeding definition and complication management. More recent data indicate that neither antithrombotic prophylaxis nor the continuation of antiplatelet therapy after KT lead to an increase in incidents of bleeding in the perioperative period, except for postoperative heparin infusion [40,41]. Initiation of heparin infusion in a therapeutical dose within 24 h after surgery has been associated with an increased risk of bleeding; accordingly, in our current analysis the bleeding risk was increased when heparin was started within the first 6 h postoperatively, whereas the prophylactic use of heparin is reported to be safe [5,6,8]. Interestingly, the use of intraoperative heparin as well as APT per se or in combination with prophylactic doses of heparin were not associated with an increased bleeding risk, which is also underlined by previous studies [5,39]. Hence, according to common clinical practice, heparin seems a good and safe choice for perioperative anticoagulation, whereas the timing and dose of postoperative heparin must be considered with caution.

Aside from antithrombotic and VKA therapy, often indicated in certain cardiovascular diseases, CVD was associated with an increased risk for bleeding, most likely explained by the technical challenges of transplanting patients with high burdens of CVD [4,5]. In accordance with previous reports, our study found a few key recipient-, procedure- and donor-specific risk factors for postoperative bleeding following KT. In this context, donor type was a strong predictor in multivariable-adjusted analyses with the risk of the development of postoperative bleeding in our cohort. This finding is most likely influenced by both graft and recipient impact. For one, surgical proceedings in graft removal differ significantly between living and deceased donors, with en bloc explantation including perinephric and retroperitoneal tissues in the latter, and a cautious preparation of the isolated kidney and the protection of all vital structures including considerate hemostasis in the former [4,26]. The probability of bleeding may be enhanced precisely due to the presence of vascularized perinephric and hilar tissues, with surgical hemostasis being only accomplishable after in situ reperfusion after the implantation of the organ. Moreover, deceased donor transplants may be more prone to bleeding episodes compared to living kidney donations due to several factors, including increased systemic anticoagulation before organ removal, advanced age, an increased number of comorbidities and differences in body habitus with thereby an augmented fragility of deceased organ donations. Furthermore, recipients of deceased organs display significantly longer periods on pretransplantation waiting lists compared to living donor kidney transplant recipients. Therefore, disadvantages on the recipient side due to prolonged dialysis and consequent adverse effects on tissue and vessel integrity may also augment the likelihood of bleeding in this cohort [31].

Interestingly, in our current study, increased BMI was strongly associated with decreased postoperative bleeding risk following renal transplant in multivariable analysis. Research in the current literature shows conflicting evidence concerning increased BMI and perioperative risk profile. In some investigations, high BMI seems to be associated with an increased risk of intraoperative and postoperative bleeding, wound healing disorder and cardiac complications [42,43,44]. However, other studies show opposite results, also referred to as the obesity paradox, with improved outcomes in patients with increased BMI [4,5,45]. Several explanations may be listed supporting our current findings: The increased tissue in high BMI patients may have worked as a natural tamponade and effectively stopped postoperative bleeding episodes in their beginning. Further, the increased body weight may have led to slightly lower effective doses of anticoagulative drugs per kg body weight due to the concern of overdosage in this patient collective. Aside from that, one must consider that the technical difficulties of conducting ultrasounds in obese patients may have led to overlooking smaller hematomas. However, the superficial location of the allograft in combination with technical advances in ultrasound visualization makes this problem rather unlikely. 

Concerning graft and patient outcome after kidney transplantation, our current study demonstrates a close relationship between postoperative bleeding episodes and increased HAS-BLED scores, leading to inferior long-term graft outcomes with increased graft failure or death. Although the precise mechanisms and pathophysiological changes are not fully understood, the mechanical compression of the graft due to the development of a hematoma as well as arterial hypotension and consecutive graft hypoperfusion may likely play major roles in delayed graft function and the deterioration of long-term outcomes. Moreover, a sudden onset of anemia and the successive need of red blood cell transfusion, in some cases disimproved by additional sepsis due to infected hematoma, may also have a significant negative impact on graft function and patient outcome. Of note, hematomas with indication for surgical interventions, such as reoperation and surgical hematoma evacuation, or interventional repairs, such as the placement of additional drainage, potentially combined with an injection of a contrast medium prior to medical CT imaging, presumably will lead to impaired long-term outcomes, as observed in our supplementary analysis by bleeding severity [4]. Other recipient-associated factors, such as an increased number of comorbidities with the need for anticoagulative drugs and a prolonged time on dialysis with negative effects on vessel and tissue integrity, may also increase the risk of long-term graft loss or death. Accordingly, in our study, the univariable association between bleeding and long-term outcomes was more accentuated in deceased versus living donor kidney transplants, due to several factors, as discussed earlier (e.g., an increased number of comorbidities, increased need for anticoagulative drugs, longer time on the waiting list before transplantation, impaired vessel integrity of donor and recipient, increased age and a higher number of renal artery reconstructions in deceased versus living donor kidney recipients).

However, due to our small study collective, these interesting preliminary findings must be re-evaluated in larger, prospective studies with sufficient statistical power and the possibility of multivariable adjustment.

Despite the many strengths of our study, a few limitations must be addressed.

Firstly, the low number of patients in each group and the retrospective nonrandomized design of our current study must be considered. Due to its retrospective design and the rather small number of patients included in the analyses, and specifically per analysis group, the results of the study should be interpreted with caution: while direct translation into clinical practice is self-evidently prohibited due to potentially low statistical power and a lack of shown causal relation, although adjustments were made for some confounders, residual confounding effects cannot be ruled out completely and the purpose and value of retrospective trials is primarily the generation of hypotheses that need to be tested in prospective trials afterwards. 

Secondly, in comparison to previous studies analyzing bleeding events following KT, our observed incidence of postoperative bleeding cases of almost 29% is ranking in the upper range of bleeding incidents according to the literature. As a possible explanation, one must discuss the relatively low number of kidney transplantations per year at our center, which may, according to current data, influence short- and long-term outcomes in solid organ transplantation procedures [46,47,48,49]. However, our observed incidence may be overestimated compared to similar studies. Firstly, our retrospectively defined criteria of postoperative bleeding were deliberately defined rather broadly to avoid the underestimation of bleeding events. Secondly, in our current study the majority of the investigated patients received (sub)therapeutic antithrombotic medication due to serious comorbidities, resulting in significantly more bleeding episodes in this group. Thirdly, in a considerable number of patients assigned to the bleeding group (nine; 5%), the postoperatively observed drop in hemoglobin may in part also have been influenced by an increased perioperative fluid challenge. On the other hand, due to the diligent conduction of daily ultrasound investigations at our center, we assume to have a very high rate of detection of even small fluid collections suspect of hematomas, which may have been overlooked in other centers and therefore confound previous results in the literature. Additionally, it must be discussed that ultrasounds are operator-variable and some ultrasounds did not definitively indicate whether a larger collection of fluid was in fact a hematoma, which may also augment findings of bleeding in our retrospective study. As an alternative to the ultrasound technique, CT scans may be analyzed to confirm the identity of suspect fluid collections, although these tests are not routinely conducted among patients at our center due to the obvious adverse side effects of radiation and contrast agents. 

Since the mentioned cases remained stable without any treatment, such as interventions/reoperation or blood infusion, in combination with stable and nonchanging fluid collections in the ultrasound, these findings could underline our thesis of a retrospective overestimation of bleeding incidents.

Thirdly, regarding the evaluation of the HAS-BLED score in KT, in our current study fewer patients received vitamin K antagonist or DOAC therapy compared to the original evaluation of the score, which may result in difficulties when drawing any strong conclusions about the predictive value of the HAS-BLED score—and resulting events in this patient group. A further validation of the predictive value of this risk score and of our retrospective findings for bleeding and graft survival in large prospective multicenter studies of patients under vitamin K antagonists/DOACs undergoing KT is surely necessary before extrapolating our results to clinical practice.

## 5. Conclusions

To date, there is still a lot of uncertainty about the adequate management of peri- and postoperative antithrombotic and antiplatelet therapy for the prevention of bleeding and thromboembolic complications following KT. Our study indicates that the HAS-BLED risk score may be a helpful tool in the risk assessment and optimization of perioperative anticoagulation management, with an adequate prognostic accuracy in KT recipients to predict bleeding events and allograft failure. Further, we observed that different antithrombotic regimes, including continued VKA therapy, supratherapeutic postoperative heparin and a combination of antiplatelet therapy with therapeutical heparin, were associated with the greatest risk of postoperative bleeding. On the other hand, the use of intraoperative heparin, postoperative APT or a combination of prophylactic heparin with APT did not increase the incidence of bleeding after KT, indicating these agents as safe options to prevent early postoperative TEC. We further show that the initiation of anticoagulation < 6 h and the administration of antiplatelet therapy within 24 h after KT are further significant predictors of the development of postoperative bleeding. Therefore, the optimal timepoint of anticoagulation or/and antiplatelet readministration as well as a critical evaluation of the vital indications of the reintroduction of anticoagulants/antiplatelet therapy after kidney transplantation are essential for outcome improvement. The associated independent negative effect of bleeding episodes on allograft survival underscores the need to reduce any risk factors for postoperative bleeding. The evaluation of the HAS-BLED score in KT may aid in identifying high-risk patients for postoperative hemorrhage and hence reduce the risk for avoidable incidents of bleeding during the early postoperative period. Large and prospective clinical multicenter studies are warranted to determine, for instance, the underlying mechanisms by which postoperative hemorrhage in kidney transplant patients leads to worse long-term clinical outcomes as well as to re-evaluate critical indications for early postoperative anticoagulation despite an increased risk of bleeding.

## Figures and Tables

**Table 1:** Baseline characteristics of the study population stratified according to antithrombotic regime.

Variables	Post-Surgical Anticoagulant Strategy		*p*-Value
	Prophylactic Anticoagulation (n = 108 Patients)	(Sub)therapeutic and/or Antiplatelet Therapy (n = 96 Patients)	
**Recipient characteristics**			
Gender (%)			
male/female	67 (62)/41 (38)	61 (64)/35 (36)	0.824
Age (years)	51.29 ± 13.13	55.88 ± 13.71	<0.05 *
BMI (kg/m²)	25.75 ± 4.18	25.68 ± 4.58	0.907
ASA score	2.99 ± 0.96	3.00 ± 0.145	0.501
Prior transplant loss (%)	8 (7)	8 (8)	0.806
Pre-emptive KT (%)	11 (10)	3 (3)	<0.05 *
Type of dialysis (%)			
Hemodialysis/CAPD	91 (94)/6 (6)	91 (98)/2 (2)	0.166
Time on dialysis (years)	5.53 ± 3.29	5.72 ± 4.13	0.721
Arterial hypertension (%)	93 (86.1)	88 (92)	0.210
Diabetes mellitus (%)	11 (10)	16 (17)	0.173
Cardiovascular disease (%)	22 (20)	59 (62)	<0.01 **
Pulmonary disease (%)	5 (5)	10 (10)	0.114
HAS-BLED score	2.27 ± 0.69	3.29 ± 1.08	<0.01 **
**Donor characteristics**			
Gender (%)			
male/female	50 (46)/58 (54)	52 (54)/44 (46)	0.216
Donor age (years)	49.8 ± 19.3	54.5 ± 16.0	0.069
Donor BMI (kg/m^2^)	29.9 ± 8.2	26.3 ± 5.8	0.245
Donor type (%)			
living/deceased	28 (26)/80 (74)	23 (24)/73 (76)	0.746
**Cause of end-stage renal disease**			
Diabetic nephropathy (%)	2 (2)	9 (9)	0.405
Hypertensive kidney disease (%)	16 (15)	11 (12)	
Primary/secondary glomerulonephritis (%)	47 (44)	40 (42)	
Polycystic kidney disease (%)	19 (18)	16 (17)	
Tubulointerstitial nephritis (%)	6 (6)	8 (8)	
Cirrhosis of kidney (%)	9 (8)	5 (5)	
Reflux nephropathy (%)	2 (2)	3 (3)	
Alport syndrome (%)	3 (3)	1 (1)	
Unknown (%)	4 (4)	3 (3)	
**Preoperative factors**			
Creatinine (mmol/L)	0.64 ± 0.21	0.66 ± 0.23	0.520
Urea (mmol/L)	15.58 ± 7.21	15.84 ± 6.57	0.798
Hemoglobin (mmol/dL)	6.89 ± 0.88	6.83 ± 0.92	0.651
Platelets (GPT/L)	206 ± 70	201 ± 66	0.561
Quick (%)	102.39 ±12.80	96.53 ± 17.98	<0.01 **
INR	0.97 ± 0.1	1.02 ± 0.24	0.054
**Immunosuppression**			
ABO incompatibility (%)	11 (11)	8 (9)	0.638
Prior induction therapy in case of HLA-mismatch (%)	71 (66)	74 (77)	0.075
Basiliximab (%)	65 (60)	68 (71)	0.111
ATG (%)	7 (7)	9 (9)	0.443
Rituximab (%)	6 (6)	5 (5)	0.913
HLA mismatch (%)	71 (66)	74 (77)	0.075

Data are shown as mean ± standard deviation (SD). BMI, body mass index; ASA, American association of anesthesiologists; KT, kidney transplantation; CAPD, continuous ambulatory peritoneal dialysis; GPT, gigaparticles per litre; INR, international normalized ratio; HLA, human leukocyte antigen; ATG, antithymoctye globulin; Intergroup comparison: prophylactic anticoagulation versus (sub)therapeutic and/or antiplatelet therapy; *p* < 0.05 *; *p* < 0.01 **.

**Table 2:** Baseline characteristics of the study population stratified according to the bleeding status.

Variables	Postoperative Bleeding Complication		*p*-Value
	No (n = 143 Patients)	Yes (n = 61 Patients)	
**Recipient characteristics**			
Gender (%)			
male/female	93 (65)/50 (35)	35 (57)/26 (43)	0.300
Age (years)	53.12 ± 13.90	54.21 ± 13.09	0.600
BMI (kg/m²)	25.66 ± 4.45	25.84 ± 4.18	0.788
ASA score	2.99 ± 0.15	3.00 ± 0	0.708
Prior transplant loss (%)	11 (8)	5 (8)	0.902
Pre-emptive KT (%)	12 (8)	2 (3)	0.182
Type of dialysis (%)			
Hemodialysis/CAPD	126 (88)/5 (4)	56 (91.8)/3 (5)	0.687
Time on dialysis (years)	5.47 ± 3.60	5.96 ± 3.98	0.399
Dialysis prior to transplant/no dialysis prior to transplant	22 (15)/121 (85)	8 (13)/53 (87)	0.674
HAS-BLED score	2.50 ± 0.92	3.44 ± 1.19	<0.01 **
Arterial hypertension (%)	125 (87)	56 (92)	0.364
Diabetes mellitus (%)	19 (13)	8 (13)	0.971
Cardiovascular disease (%)	47 (33)	34 (56)	<0.01 **
Pulmonary disease (%)	12 (8)	3 (5)	0.384
**Donor characteristics**			
Donor gender (%)			
male/female	67 (47)/76 (53)	35 (87)/26 (13)	0.162
Donor BMI (kg/m²)	25.3 ± 4.2	29.8 ± 7.2	0.533
Donor age (years)	54.6 ± 17.5	59.9 ± 18.2	0.193
Donor type (%)			
living/deceased	42 (29)/101 (71)	9 (15)/52 (85)	<0.05 *
**Cause of end-stage renal disease**			
Diabetic nephropathy (%)	7 (5)	4 (7)	0.498
Hypertensive kidney disease (%)	17 (12)	10 (16)	
Primary/secondary glomerulonephritis (%)	67 (47)	20 (33)	
Polycystic kidney disease (%)	23 (16)	12 (20)	
Tubulointerstitial nephritis (%)	7 (5)	7 (12)	
Cirrhosis of kidney (%)	11 (8)	3 (5)	
Reflux nephropathy (%)	4 (3)	1 (2)	
Alport syndrome (%)	2 (1)	2 (3)	
Unknown (%)	5 (4)	2 (3)	
**Preoperative factors**			
Prior anticoagulation (%)			
Prior antiplatelet drug	47 (33)	26 (43)	0.183
Prior plasmatic anticoagulant/VKA therapy	8 (6)	10 (16)	<0.05 *
Postoperative therapeutical heparin (%)	20 (14)	23 (37)	<0.01 **
Intraoperative heparin (%)	9 (6)	8 (13)	0.102
Creatinine (mmol/L)	0.64 ± 0.22	0.67 ± 0.24	0.457
Urea (mmol/L)	15.79 ± 7.15	15.49 ± 6.36	0.784
GFR (ml/min)	8.23 ± 3.34	7.79 ± 3.41	0.392
Hemoglobin (mmol/L)	7.51 ± 0.90	7.48 ± 0.91	0.812
Platelets (GPT/L)	230 ± 71	203 ± 58	<0.05 *
INR	0.98 ± 0.12	1.02 ± 0.24	0.125
Quick (%)	108 ± 16	105 ± 20	0.159
PTT (s)	31.6 ± 20.4	30.8 ± 5.3	0.776
**Immunological factors**			
ABO incompatibility (%)	13 (9)	6 (10)	0.927
Prior induction therapy in case of HLA-mismatch (%)	100 (70)	45 (74)	0.156
Basiliximab (%)	92 (64)	41 (67)	0.693
ATG (%)	9 (6)	7 (12)	0.208
Rituximab (%)	6 (4)	5 (8)	0.247

Data are shown as mean ± standard deviation (SD). BMI, body mass index; ASA, American association of anesthesiologists; KT, kidney transplantation; CAPD, continuous ambulatory peritoneal dialysis; VKA, vitamin K antagonists; GFR, glomerular filtration rate; GPT, gigaparticles per litre; INR, international normalized ratio; PTT, partial thromboplastin time; HLA, human leukocyte antigen; ATG, antithymoctye globulin; Intergroup comparison: no bleeding versus bleeding; *p* < 0.05 *; *p* < 0.01 **.

**Table 3:** Intra- and postoperative characteristics of our study population according to antithrombotic medication regime.

	Postsurgical Anticoagulant Strategy		*p*-Value
Variables	Prophylactic Anticoagulation (n = 108)	(Sub)therapeutic and/or Antiplatelet Therapy (n = 96)	
Number of arteries			
mean ± SD	1.09 ± 0.32	1.17 ± 0.45	0.183
1/>1 (%)	98 (92)/9 (8)	82 (86)/13 (14)	0.230
Number of veins			
mean ± SD	1.06 ± 0.23	1.05 ± 0.22	0.918
1/>1 (%)	101 (94)/6 (6)	90 (95)/5 (5)	0.914
Cold ischemia time (min)	532 ± 329	523 ± 299	0.859
Warm ischemia time (min)	46 ± 23	43 ± 17	0.519
Surgery time (min)	187 ± 41	200 ± 64	0.098
Blood loss (mL)	133 ± 212	192 ± 387	0.193
Kidney right/left	58 (54)/50 (46)	46 (47)/50 (53)	0.591
Total transfusion during surgery (mL)	2943.18 ± 1205.41	3385 ± 1554.64	<0.05 *
Crystalloids (mL)	2892.15 ± 1167.77	3162.19 ± 1288.93	0.119
Crystalloids (mL/kg)	38.89 ± 16.48	43.31 ± 19.38	0.820
Crystalloids (mL/kg/h)	12.93 ± 5.60	13.62 ± 6.97	0.446
Red blood cells (mL)	42.06 ± 167.13	159.38 ± 541.84	<0.05 *
FFP (mL)	46.17 ± 63.80	57.29 ± 325.91	0.134
PLT (mL)	2.80 ± 29.00	9.38 ± 52.47	0.264
Postreperfusion urine output, 1 h (mL)	162 ± 92	68 ± 101	<0.05 *
Diuresis day 1 (mL)	1961 ± 1290	1240 ± 1100	<0.01 **
Delayed graft function (%)	17 (16)	37 (39)	<0.01 **
Rejection of transplant organ (%)	29 (28)	36 (38)	0.098
During hospitalization/≤12/>12 months	14 (13)/7 (7)/8 (7)	17 (18)/11 (12)/8 (8)	0.806
Thromboembolic complications (%)	3 (3)	8 (8)	0.08
Bleeding complication (%)	21 (10)	40 (20)	<0.01 **
Complications according to Clavien–Dindo classification			
minor/major (%)	70 (65)/38 (35)	38 (40)/58 (60)	<0.05 *
Platelets day 1 (GPT/L), mean ± SD	195 ± 63	189 ± 68	0.531
Quick day 1 (%), mean ± SD	101 ± 15	94 ± 17	<0.01 **
PTT day 1 (s), mean ± SD	27 ± 5.3	38 ± 21	<0.01 **
Platelets day 3 (GPT/L), mean ± SD	187 ± 69	182 ± 66	0.571
Quick day 3 (%), mean ± SD	112 ± 15	101 ± 15	0.09
PTT day 3 (s), mean ± SD	26 ± 8	37 ±19	<0.01 **
Platelets day 5 (GPT/L), mean ± SD	210 ± 88	193 ±71	0.216
Quick day 5 (%), mean ± SD	113 ± 15	105 ± 16	<0.01 **
PTT day 5 (s), mean ± SD	26 ± 3.5	36 ± 19	<0.01 **

Data are shown as mean ± standard deviation (SD). FFP, fresh frozen plasma; PLT, platelets; GPT, gigaparticles per litre; PTT, partial thromboplastin time; s, seconds; Intergroup comparison: prophylactic anticoagulation versus (sub)therapeutic and/or antiplatelet therapy; *p* < 0.05 *; *p* < 0.01 **.

**Table 4:** Intra- and postoperative outcomes of the study population according to bleeding status.

Variables	Postoperative Bleeding Complication		*p*-Value
	No (n = 143)	Yes (n = 61)	
Number of arteries			
mean ± SD	1.15 ± 0.41	1.08 ± 0.33	0.245
1/>1 (%)	124 (87)/18 (13)	56 (92)/4 (7)	0.210
Number of veins			
mean ± SD	1.05 ± 0.22	1.07 ± 0.25	0.621
1/>1 (%)	135 (94)/7 (5)	56 (92)/4 (7)	0.619
Cold ischemia time (min), mean ± SD	544 ± 325	492 ± 290	0.311
Warm ischemia time (min), mean ± SD	43 ± 21	49 ± 20	0.157
Surgery time (min), mean ± SD	186 ± 41	210 ± 72	<0.05 *
Blood loss (mL), mean ± SD	113 ± 164	274 ± 488	<0.05 *
Kidney right/left (%)	71 (50)/72 (50)	33 (54)/28 (66)	0.564
Total transfusion during surgery (mL), mean ± SD	2936.06 ± 1085.08	3651.64 ± 1848.37	<0.01 **
Crystalloids (mL), mean ± SD	2933.94 ± 1082.40	3219.84 ± 1513.19	0.130
Crystalloids (mL/kg), mean ± SD	39.76 ± 15.34	43.76 ± 22.90	0.148
Crystalloids (mL/kg/h), mean ± SD	13.23 ± 5.86	13.33 ± 7.62	0.915
Red blood cells (mL), mean ± SD	2.11 ± 25.18	319.67 ± 672.76	<0.01 **
FFP (mL), mean ± SD	0.00 ± 0.00	100.98 ± 412.71	0.061
PLT (mL), mean ± SD	0.00 ± 0.00	19.67 ± 74.88	<0.05 *
Postreperfusion urine output, 1 h (mL)	101.17 ± 165.60	58.61 ± 126.18	<0.05 *
Diuresis day 1 (mL)	1791.05 ± 1730.84	1214.20 ± 1213.93	<0.05 *
Delayed graft function (%)	31 (22)	23 (38)	<0.05 *
Rejection of transplant organ (%)	37 (26)	28 (46)	0.426
During hospitalization/≤12/>12 months	17 (12)/11 (8)/9 (6)	14 (23)/7 (12)/7 (12)	0.911
Thromboembolic complications (%)	4 (3)	7 (11)	<0.05 *
Complications according to Clavien–Dindo classification			
minor/major (%)	93 (6)/50 (35)	15 (24)/46 (76)	<0.01 **
Platelets day 1 (GPT/L), mean ± SD	198 ± 63	178 ± 57	<0.05 *
Quick day 1 (%), mean ± SD	98 ± 16	96 ± 17	0.437
PTT day 1 (s), mean ± SD	29 ± 9	37 ± 22	<0.01 **
Platelets day 3 (GPT/L), mean ± SD	192 ± 66	167 ± 68	<0.05 *
Quick day 3 (%), mean ± SD	110 ±13	109 ± 19	0.826
PTT day 3 (s), mean ± SD	28 ± 13	36 ± 19	<0.05 *
Platelets day 5 (GPT/L), mean ± SD	213 ± 80	176 ± 71	<0.01 **
Quick day 5 (%), mean ± SD	109 ± 14	108 ± 17	0.908
PTT day 5 (s), mean ± SD	30 ± 15	35 ± 18	0.233

Data are shown as mean ± standard deviation (SD). FFP, fresh frozen plasma, PLT, platelets; GPT, gigaparticles per litre; PTT, partial thromboplastin time; s, seconds; Intergroup comparison: no bleeding versus bleeding; *p* < 0.05 *; *p* < 0.01 **.

**Table 5:** Multivariable logistic regression analysis for risk factors for bleeding following kidney transplantation.

Variables	Odds Ratio (95% CI)	*p*-Value
**Recipient characteristics**		
Recipient BMI (per 5 kg/m^2^ increase)	0.32 (0.12–0.91)	<0.05 *
Time on dialysis pretransplant (per 1-year increase)	1.08 (0.92–1.18)	0.165
Recipient peripheral arterial disease (yes versus no)	1.36 (0.42–3.25)	0.235
Recipient cardiovascular disease (yes versus no)	2.89 (1.02–8.21)	<0.05 *
HAS-BLED score	1.49 (1.08–2.07)	<0.05 *
HAS-BLED score > 2	5.41 (1.21–24.16)	<0.05 *
**Donor characteristics**		
Donor age, >55 years	1.02 (0.99–1.05)	0.11
Donor BMI (per 5 kg/m^2^ increase)	1.049 (0.92–1.19)	0.471
Donor type (living versus deceased)	0.43 (0.18–0.94)	<0.05 *
**Transplant characteristics**		
Blood loss, mL	1.03 (1.0–1.05)	<0.05 *
Delayed graft function (yes versus no)	1.92 (0.98–3.77)	0.056
Clavien–Dindo classification > grade II	3.34 (1.17–9.49)	<0.05 *
Cold ischemia time, hours	0.99 (0.99–1.00)	0.09
Anastomosis time, min	1.01 (0.98–1.05)	0.256
Duration of surgery, hours	0.99 (0.98–1.06)	0.474
**Pharmacological and laboratory characteristics**		
Preoperative VKA therapy	5.89 (1.10–31.28)	<0.05 *
Preoperative antiplatelets (yes versus no)	1.63 (0.85–3.15)	0.144
Intraoperative heparin	1.35 (0.44–4.11)	0.597
Postoperative heparin	2.5 (1.1–5.69)	<0.05 *
Postoperative antithrombotic regime ((sub)therapeutic versus prophylactic)	2.14 (1.08–4.24)	<0.05 *
Prophylaxis	1.0	
Antiplatelet + prophylactic heparin	0.998 (0.39–2.52)	0.996
Therapeutic heparin	3.37 (1.37–8.23)	<0.01 **
Antiplatelet + therapeutic heparin	5.44 (1.31–22.31)	<0.05 *
Start antithrombotic therapy <6 h versus >6 h	1.58 (0.85–2.98)	0.151
Starts platelets within 24 h versus after KT	1.57 (0.71–3.49)	0.268

BMI, body mass index; min, minutes; VKA, vitamin K antagonists; KT, kidney transplantation; min, minutes; *p* < 0.05 *; *p* < 0.01 **.

**Table 6:** Cox regression analysis of clinically and paraclinically relevant factors correlated to graft loss and/or death following kidney transplantation.

**Variables**	**Univariate**		**Multivariate**	
	***HR (95% CI)***	***p-Value***	***HR (95% CI)***	***p-Value***
**Recipient characteristics**				
Recipient age, years	1.06 (1.01–1.10)	<0.01 **	NS	NS
Recipient gender (male versus female)	2.08 (0.94–4.6)	0.06		
Recipient BMI > 30 kg/m^2^ versus <18.5–24.9 kg/m^2^	1.96 (1.18–3.07)	<0.05 *	NS	NS
Type of transplantation (pre-emptive versus not)	2.8 (1.3–5.2)	<0.05 *	NS	NS
Recipient history of diabetes (yes versus no)	1.99 (1.02–3.12)	<0.05 *	1.71 (1.07–2.73)	<0.05 *
Recipient cardiovascular disease (yes versus no)	1.99 (1.01–3.91)	<0.05 *	NS	NS
HAS-BLED score, >3 versus <3	4.01 (1.87–8.90)	<0.01 **	3.2 (1.46–7.1)	<0.01 **
**Donor characteristics**				
Donor age, <55 versus >55 years	2.8 (1.3–5.9)	<0.01 **	3.2 (1.5–6.81)	<0.01 *
Donor type (living versus deceased)	11.6 (1.55–83.1)	<0.01 **	NS	NS
**Transplant characteristics**				
Cold ischemia time, hours	1.01 (1.00–1.02)	<0.05 *	NS	NS
Duration of surgery, hours	1.007 (1.003–1.011)	<0.01 **	1.006 (1.002–1.01)	<0.01 **
Acute rejection	3.2 (1.6–5.4)	<0.01 **	2.71 (1.83–4.1)	<0.01 **
Delayed graft function (yes versus no)	2.47 (1.25–4.87)	<0.01 **	NS	NS
Clavien–Dindo score, <2 versus >2	4.2 (1.7–10.3)	<0.01 **	4.2 (1.7–9.4)	<0.01 **
**Pharmacological factors (preoperative, during hospital stay and follow-up)**				
Preoperative anticoagulation (yes versus no, not stopped or INR not corrected)	3.35 (1.01–10.93)	<0.05 *	5.21 (1.4–18.9)	**<0.05**
Preoperative antidiabetics (yes versus no)	3.12 (1.05–9.21)	<0.05 *	NS	NS
Antithrombotic therapy postoperative ((sub) therapeutic versus prophylactic)	3.32 (1.6–6.9)	<0.01 **	2.9 (1.41–6.23)	<0.01 **
Transfusion blood products postop (yes versus not)	2.57 (1.11–5.46)	<0.05 *	NS	NS
Postoperative catecholamine therapy (yes versus not)	5.51 (2.67–11.3)	<0.01 **	3.79 (1.4–6.8)	<0.01 **
Antibiotics postoperative (yes versus not)	3.3 (1.64–6.67)	<0.01 **	NS	NS
Platelet inhibitor postoperative	2.91 (1.4–5.72)	<0.01 **	NS	NS
CMV infection/CMV therapy (yes versus no)	1.59 (1.13–2.2)	<0.05 *	NS	NS
Tranexamic acid	1.01 (1.001–1.003)	<0.01 **	NS	NS
Change in immunosuppression (yes versus no)	4.32 (1.23–15.18)	<0.05 *	NS	NS
Treatment for poliomavirus (change IS, immunoglobulin)	3.49 (1.62–7.2)	<0.01 **	NS	NS
Fungal therapy	4.2 (1.6–10.9)	<0.05 *	NS	NS
Postoperative antithrombotic regime		<0.01 **		<0.05 *
Prophylaxis	1.0		1.0	
Platelet + prophylactic heparin	2.91 (1.03–8.18)	<0.05 *	2.8 (1.1–7.4)	0.123
Platelet + therapeutical Heparin	4.23 (1.82–9.81)	<0.01 **	3.3 (1.52–6.58)	<0.05 *
Postoperative heparin (therapeutically)	6.76 (2.26–20.56)	<0.01 **	2.25 (0.82–6.13)	0.07

The following clinical, paraclinical and pharmacological variables were tested in univariate analysis but failed to show any significance for allograft failure: *(Para)clinical factors*: donor gender, donor body mass index, type of dialysis (hemodialysis versus peritoneal dialysis), recipient arterial hypertension, retransplantation, donation of the kidney (right versus left), AB0-incompatible KT, warm ischemia time, human leukocyte antigen (0–2 versus >3) and panel reactive antibodies. Pharmacological medications and treatments preoperatively, during hospital stay and follow-up: *General*: induction therapy, specifically induction therapy (ATG versus rituximab versus IL-2 antagonists), initial immunosuppression (CNI versus mTOR), early steroids withdrawal and intraoperative heparin use. *Preoperative*: antiplatelets, antihypertensive medications (0–2 versus >2), statins, thyroid and parathyroid medication, diuretics, analgesics, gastric inhibitors, bone protection/calcium homeostasis and vitamin D metabolism, hormones (iron medications and erythropoietin), phosphate binder, bicarbonate. *Postoperative/follow-up*: antihypertensive medications (0–2 versus >2), statins, thyroid and parathyroid medication, diuretics, analgesics, antidiabetic medications, gastric inhibitors, bone protection/calcium homeostasis and vitamin D metabolism, hormones (iron medications and erythropoietin), phosphate binder, bicarbonate, CMV prophylaxis therapy and PCP prophylaxis therapy. 95% CI, 95% confidence interval; HR, hazard ratio; NS, not significant; BMI, body mass index; CMV, cytomegalovirus; IS, immunosuppression; *p* < 0.05 *; *p* < 0.01 **.

## Data Availability

Our database contains highly sensitive data that may reveal clinical and personal information about our patients and lead to their identification. Therefore, according to organizational restrictions and regulations, these data cannot be made publicly available. However, the datasets used and/or analyzed in the current study are available from the corresponding author upon reasonable request.

## Supplementary Materials

The following supporting information can be downloaded at: https://www.mdpi.com/article/10.3390/jcm11144025/s1, Table S1: Summary of post-operative drugs/ doses stratified according to prophylaxis and (sub)therapeutic group. Table S2: Long-term graft function stratified by antithrombotic medication regime and bleeding episodes. Table S3: Univariate logistic regression analysis for risk factors for bleeding following kidney transplantation. Figure S1: Receiver operating characteristics (ROC) curve of HAS-BLED risk score for bleeding events in patients undergoing kidney transplantation. Figure S2: Kidney transplant outcome stratified by bleeding management. Figure S3: Transplant outcome according to bleeding status and donor type.

## Abbreviations

AF	atrial fibrillation
ANOVA	analysis of variance
APT	antiplatelet therapy
ARE	acute rejection episode
ASA	American Society of Anesthesiologists
ATG	Antithymoctye globulin
AUC	area under the curve
APT	antiplatelet therapy
BMI	body mass index
CDC	Clavien–Dindo classification
CI	confidence interval
CKD	chronic kidney disease
CMV	cytomegalovirus
CT	computed tomography
CVD	cerebrovascular disease
DGF	delayed graft function
DOAC	direct oral anticoagulants
EC	erythrocyte concentration
ERSD	end-stage renal disease
FFP	fresh frozen plasma
GFR	glomerular filtration rate
HLA	human leukocyte antigen
HLT	Hosmer–Lemshow test
HR	hazard Ratio
INR	international normalized ratio
KT	kidney transplantation
OR	odds ratio
PAD	peripheral arterial disease
PTT	partial thromboplastin time
ROC	receiver operating characteristics
TEC	thromboembolic complications
UFH	unfractionated heparin
VKA	vitamin K antagonists
VTE	venous thromboemolism
