# Supplementary material for: Predictive Value of HAS-BLED Score Regarding Bleeding Events and Graft Survival following Renal Transplantation
Source: J Clin Med. 2022 Jul 12;11(14):4025. doi: 10.3390/jcm11144025 (PMC9319563; doi:10.3390/jcm11144025)

Supplementary information

Table S1: Summary of post-operative drugs/ doses stratified according to prophylaxis and (sub)therapeutic group

**Included postoperative drugs/doses**	**Prophylaxis group**	**(sub)therapeutic group**
Aspirin	Not administered	100 mg 1-0-0
Clopidogrel/Plavix	Not administered	75 mg 1-0-0
Unfractionated Heparin	5000 UE s.c. (1-1-1)<br><br>**Or**<br><br>200 – 600 IU/h (< 15 IU/kg/h, perfusor)	Perfusor: ≥ 15 IU/ kg/ h (starting dosage, then PTT controlled, target: 60 – 80s)
Low molecular weight heparin	Tanzaparin (Innohep®): 0.35 ml; 3500 IU s.c. (1-0-0)	Tanzaparin (Innohep®): 175 IU/ kg s.c. (1-0-0)
	Nadroparin Fraxaparin®):<br><br>0.3ml, 2.850 I.E. 1-0-0	Nadroparin Fraxaparin®):<br><br>0.1 mL/10 kgKG s.c. 1-0-1
Argatroban (n=2, HIT history)	0.1 – 0.24µg/min/kg	Not administered
Danaparoid (n=1, HIT-history)	1500 Anti-Xa-units (loading dose) s.c.	Not administered

	750 Ant-Xa- units s.c. (1-0-1)	
Warfarin	Not administered	Not administered postoperative course; see below pre-operative regime
Direct oral anticoagulants (DOAC)	Not administered	Not administered postoperative course; see below pre-operative regime

Table S2: Long-term graft function stratified by antithrombotic medication regime and bleeding episodes

Variables	prophylactic anticoagulation (n = 108 patients)	(Sub)therapeutic and/or antiplatelet therapy (n = 96 patients)	p-value	No Bleeding (n = 143 patients)	Bleeding (n = 61 patients)	p-value
Mean serum creatinine at 1 month (mmol/l)	0.17 ± 0.82	0.19 +/- 0.13	0.213	0.18 ± 0.11	0.19 ± 0.12	0.646
Mean GFR at 1 month (ml/min)	41 ± 17	39 ± 18	0.360	41 ± 18	38 ± 18	0.382
Mean serum urea at 1 month (mmol/l)	9.49 ± 4.22	9.71 ± 4.57	0.728	9.93 ± 4.41	8.79 ± 4.23	0.098
Mean serum creatinine at 3 months (mmol/l)	0.16 ± 0.55	0.19 ± 0.17	0.055	0.16 ± 0.06	0.19 ± 0.11	0.056
Mean GFR at 3 months (ml/min)	42 ± 15	39 ± 18	0.168	42 ± 16	37 ± 17	0.059
Mean serum urea at 3 months (mmol/l)	9.68 ± 4.74	10.80 ± 5.93	0.162	9.84 ± 4.65	11.01 ± 6.62	0.231
Mean serum creatinine at 6 months (mmol/l)	0.16 ± 0.07	0.15 ± 0.08	0.776	0.15 ± 0.07	0.15 ± 0.07	0.937
Mean GFR at 6 months (ml/min)	45 ± 16	43 ± 18	0.508	45 ± 17	43 ± 16	0.634

Mean serum urea at 6 months (mmol/l)	10.03 ± 4.46	9.95 ± 4.06	0.909	10.1 ± 4.49	9.74 ± 3.76	0.631
Mean serum creatinine at 9 months (mmol/l)	0.14 ± 0.04	0.17 ± 0.13	0.144	0.15 ± 0.07	0.17 ± 0.14	0.300
Mean GFR at 9 months (ml/min)	47 ± 18	42 ± 18	0.175	46 ±19	42 ± 17	0.302
Mean serum urea at 9 months (mmol/l)	9.79 ± 4.51	10.95 ± 5.37	0.176	10.05 ± 4.73	10.95 ± 5.41	0.331
Mean serum creatinine at 12 months (mmol/l)	0.15 ± 0.06	0.17 ± 0.12	0.214	0.16 ± 0.08	0.17 ± 0.12	0.461
Mean GFR at 12 months (ml/min)	47 ± 18	43 ± 18	0.194	46 ± 18	44 ± 18	0.537
Mean serum urea at 12 months (mmol/l)	15.36 ± 49.59	11.01 ± 6.76	0.474	14.82 ± 43.94	10.04 ± 4.64	0.469

Data shown as median +/- standard deviation (SD); GFR, glomerular filtration rate;

Inter-group comparison: no bleeding versus bleeding and prophylactic anticoagulation versus (Sub)therapeutic and/or antiplatelet therapy.

Table S3: Univariate logistic regression analysis for risk factors for bleeding following kidney transplantation

Variables	Odds ratio (95% CI)	p-value
**Recipient characteristics**		
Age, years	1.06 (0.98-1.02)	0.598
Gender (female versus male)	1.38 (0.75-2.55)	0.301
BMI (per 5 kg/m2 increase)	0.49 (0.21-0.99)	**<0.05***
BMI > 30 kg/m2	0.38 (0.15-0.95)	**< 0.05***
Time on dialysis pretransplant (per 1 year increase)	1.13 (1.06-1.22)	**< 0.05***
Type of dialysis (Hemodialysis versus CAPD)	0.44 (0.03-7.16)	0.564
Diabetes mellitus (yes versus no)	0.87 (0.76-2.3)	0.245
Peripheral arterial disease (yes versus no)	1.55 (0.76-4.23)	0.110
Cardiovascular disease (yes versus no)	2.57 (1.39-4.75)	**<0.01****
HAS-BLED Score	1.67 (1.23-2.23)	**<0.01****

ASA score, per 1 point increase	2.21 (1.04-4.89)	**< 0.01****
**Donor characteristics**		
Age, > 55 years	1.97 (1.07-3.62)	**< 0.05***
Gender (female versus male)	1.61 (0.62-1.91)	0.521
BMI (per 5 kg/m2 increase)	1.45 (0.71-3.67)	0.189
Type (living versus deceased)	0.41 (01.8-0.92)	**< 0.05***
Donation of the kidney (right versus left)	0.19 (0.16-2.17)	0.181
**Transplant characteristics**		
Blood loss, ml	1.01 (1.01-1.03)	**< 0.05***
Re-transplantation (yes versus no)	1.07 (0.35-3.2)	0.902
Number of arteries (>1 versus 1)	1.36 (0.54-3.45)	0.506
Cold ischemia time (hours)	1.05 (1.00-1.13)	0.07
Anastomosis time > 45min	0.38 (0.20-0.73)	**<0.01****
Duration of surgery, hours	1.09 (1.00-1.02)	**< 0.01 ****

Postoperative diuresis at 1 hour, ml	0.99 (0.99-1.00)	0.081
ABO- incompatible KT (yes versus no)	1.05 (0.37-2.90)	0.927
Delayed Graft function (yes versus no)	2.74 (1.45-5.08)	**< 0.01****
Rejection Episodes (yes versus no)	6.81 (2.04-22.46)	**< 0.01****
Infectious complications	6.9 (2.67-17.82)	**< 0.01****
Clavien-Dindo Classification > Grade II	8.62 (4.05-18.4)	**< 0.01****
**Pharmacological and laboratory characteristics**		
Induction Therapy (yes versus no)	1.21 (0.62-2.37)	0.580
Initial immunosuppression (CNI versus mTOR)	0.7 (0.37-1.58)	0.120
Preoperative analgetics	1.55 (0.9-2.67)	0.07
Postoperative analgetics	1.23 (0.75-2.1)	0.09
CMV prophylaxis	1.85 (0.98–3.49)	0.06
Initial platelets (every 10 unit increased)	0.97 (0.93-1.02)	0.286

Preoperative Anticoagulation (yes versus no, not stopped, INR not corrected)	6.25 (1.18-33.39)	**< 0.05***
Preoperative antiplatelets (yes versus no)	1.57 (0.82-2.81)	0.186
Intraoperative heparin	2.25 (0.82-6.13)	0.102
Postoperative antithrombotic regime ((sub) therapeutically versus prophylactic)	2.95 (1.58-5.53)	**< 0.01****
Postoperative antithrombotic regime		
Prophylaxis	1.0	
Platelet + Prophylactic heparin	1.97 (0.93-4.18)	0.08
Platelet + Therapeutical Heparin	9.24 (2.67-33.85)	**<0.01****
Postoperative Heparin (therapeutically)	3.95 (1.95-8.02)	**<0.01****
Catecholamine use	8.12 (2.9-21.9)	**< 0.01****
Start anticoagulation, hours	0.9 (0.81-0.99)	**< 0.05***
Start anticoagulation, <6 versus > 6hours	0.54 (0.29-0.99)	**< 0.05***

Antiplatelet therapy <24 hours versus > 24 hours	2.15 (1.03-4.39)	**< 0.05***
Maximum PTT-value, at day 1	1.03 (1.01-1.06)	**< 0.01****
Maximum platelets, at day 1	0.994 (0.98-0.99)	**< 0.05***
Maximum platelets, at day 3	0.94 (0.98-0.99)	**< 0.05***
Maximum PTT-value, at day 5	1.03 (1.00-1.05)	**< 0.05***
Maximum platelets, at day 5	0.99 (0.98-0.99)	**< 0.01****

The following clinical, paraclinical and pharmacological variables were tested in univariate analysis but failed to show any significance for allograft failure: *(Para)clinical factors:* antihypertensive medications (0-2 versus > 2); statins, thyroid and parathyroid medication, diuretics, gastric inhibitors, bone protection/ calcium homeostasis and Vitamin D-metabolism; hormons (iron medications, erythropoietin), phosphate binder, bicarbonate. *Postoperative/follow-up:* antihypertensive medications (0-2 versus > 2); statins, thyroid and parathyroid medication, diuretics, analgetics, antidiabetic medications, gastric inhibitors, bone protection/ calcium homeostasis and Vitamin D- metabolism; hormons (iron medications, erythropoietin), phosphate binder, bicarbonate, CMV prophylaxis and CMV therapy, PCP prophylaxis therapy.

95% CI, 95% confidence interval; NS, not significant; BMI, body mass index; ASA, American society of Anesthesiologists; CNI, calcineurin inhibitors; mTOR, mechanistic target of rapamycin; INR, international normalized ratio; KT, kidney transplantation; CMV, cytomegalovirus; PCP, pneumocystis carinii; IS, immunosuppression; PTT, partial throboplastin time; $p < 0.05$ *; $p < 0.01$ **;

Figure S1: Receiver operating characteristics (ROC) curve of HAS-BLED risk score for bleeding events in patients undergoing kidney transplantation

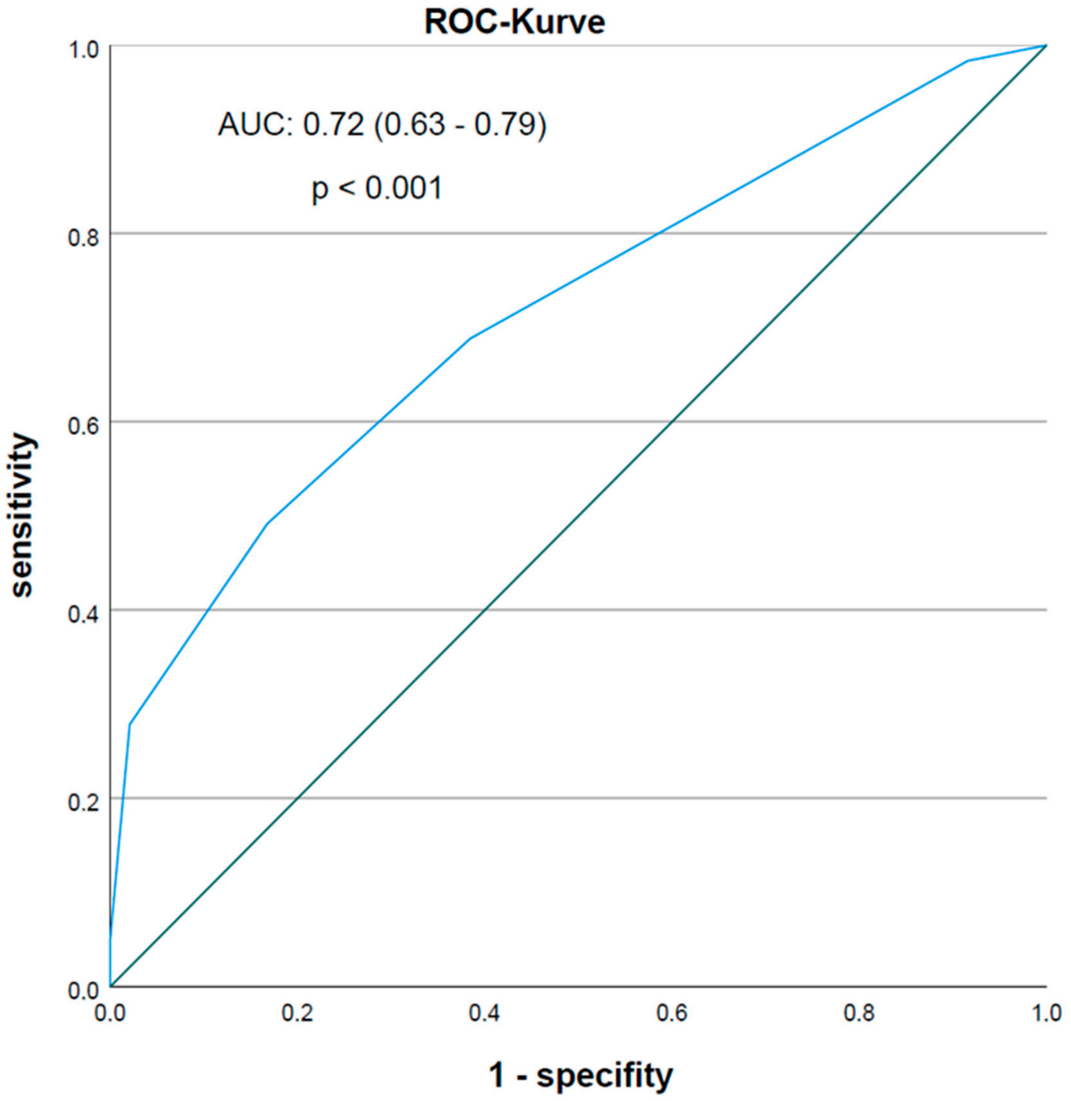

Figure S2: Kidney transplant outcome stratified by bleeding management

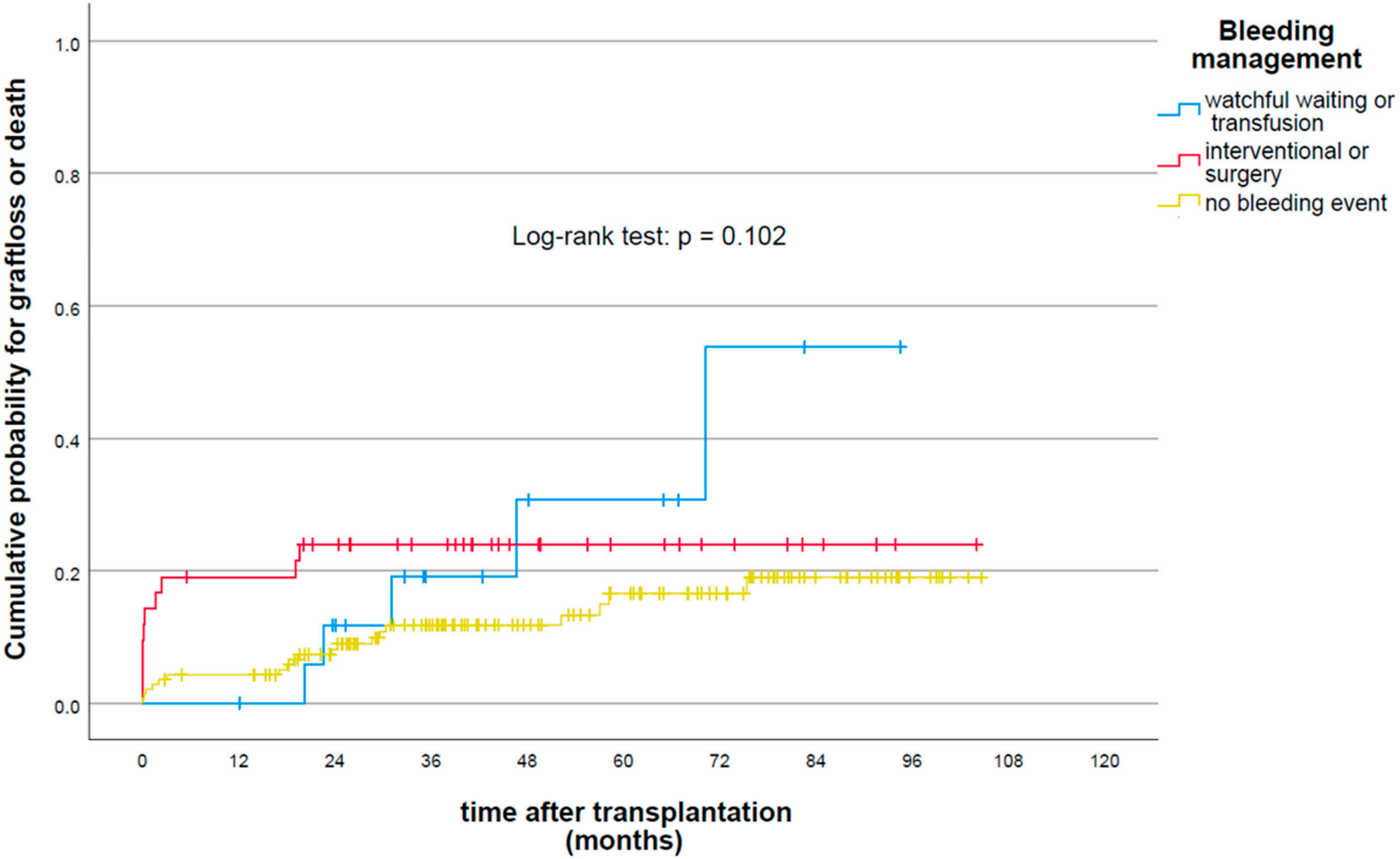

Figure S3: Transplant outcome according to bleeding status and donor type

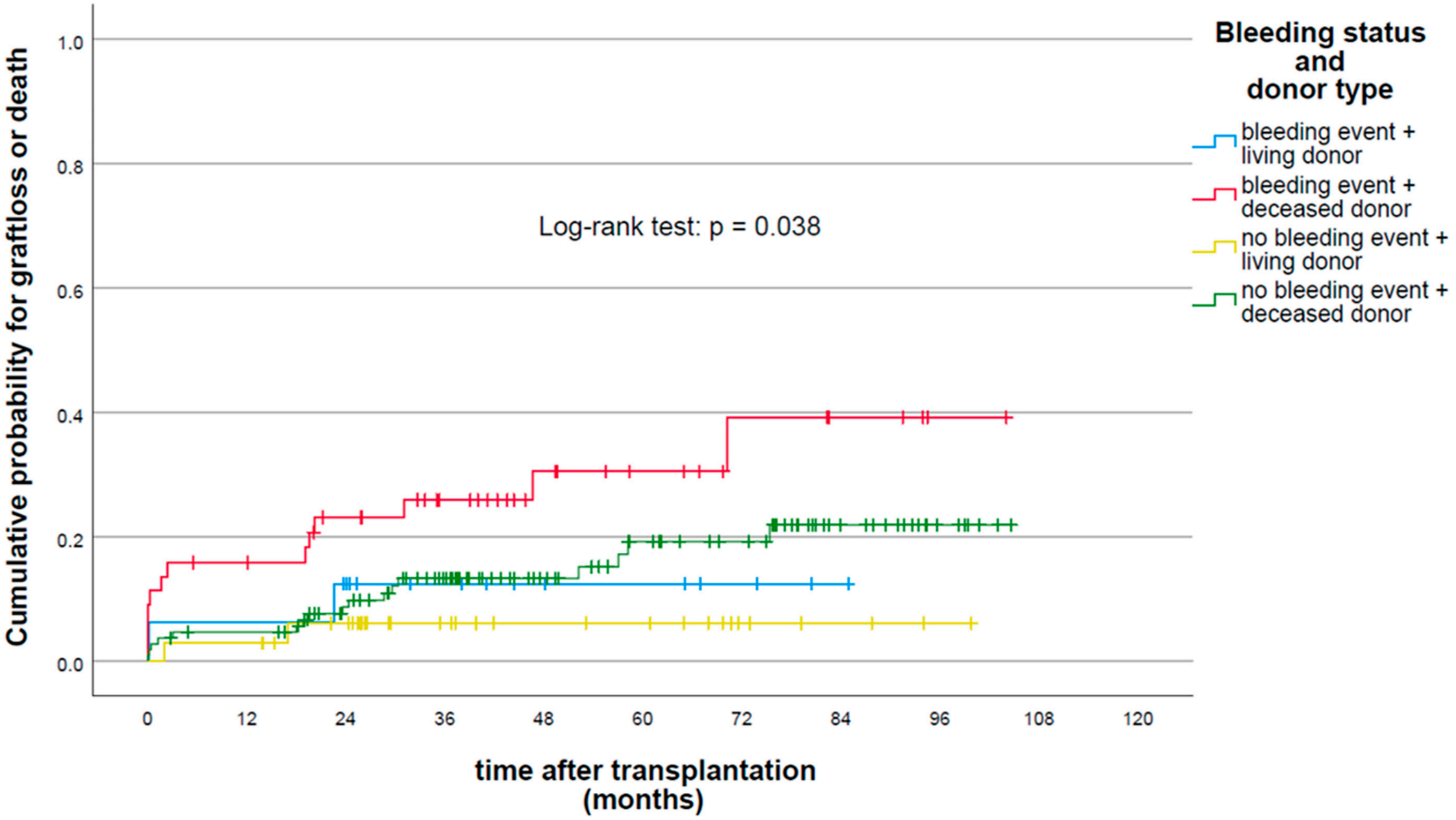

Supplement: Supplementary file 1 [file jcm-11-04025-s001.zip › jcm-1742270-supplementary.pdf]
